# Movement behavior in a dominant ungulate underlies successful adjustment to a rapidly changing landscape following megafire

**DOI:** 10.1186/s40462-024-00488-4

**Published:** 2024-07-31

**Authors:** Kendall L. Calhoun, Thomas Connor, Kaitlyn M. Gaynor, Amy Van Scoyoc, Alex McInturff, Samantha E.S. Kreling, Justin S. Brashares

**Affiliations:** 1 Department of Environmental, Science, Policy, and Management, University of California Berkeley, 137 Mulford #3114, Berkeley, CA 94720 USA; 2 Departments of Zoology & Botany, University of British Columbia, Vancouver, BC V6T 1Z4 Canada; 3 Washington Cooperative Fish and Wildlife Research Unit, School of Environmental and Forest Sciences, U.S. Geological Survey, University of Washington, Seattle, WA USA; 4 School of Environmental and Forest Sciences, University of Washington, University of Washington, Anderson Hall, Box 352100, Seattle, WA 98195 USA; 5 210 Wellman Hall, Berkeley, CA 94720 USA

**Keywords:** Megafire, Movement ecology, Black-tailed deer, Resource selection functions, Hidden Markov models, Behavioral plasticity

## Abstract

**Background:**

Movement plays a key role in allowing animal species to adapt to sudden environmental shifts. Anthropogenic climate and land use change have accelerated the frequency of some of these extreme disturbances, including megafire. These megafires dramatically alter ecosystems and challenge the capacity of several species to adjust to a rapidly changing landscape. Ungulates and their movement behaviors play a central role in the ecosystem functions of fire-prone ecosystems around the world. Previous work has shown behavioral plasticity is an important mechanism underlying whether large ungulates are able to adjust to recent changes in their environments effectively. Ungulates may respond to the immediate effects of megafire by adjusting their movement and behavior, but how these responses persist or change over time following disturbance is poorly understood.

**Methods:**

We examined how an ecologically dominant ungulate with strong site fidelity, Columbian black-tailed deer (*Odocoileus hemionus columbianus*), adjusted its movement and behavior in response to an altered landscape following a megafire. To do so, we collected GPS data from 21 individual female deer over the course of a year to compare changes in home range size over time and used resource selection functions (RSFs) and hidden Markov movement models (HMMs) to assess changes in behavior and habitat selection.

**Results:**

We found compelling evidence of adaptive capacity across individual deer in response to megafire. Deer avoided exposed and severely burned areas that lack forage and could be riskier for predation immediately following megafire, but they later altered these behaviors to select areas that burned at higher severities, potentially to take advantage of enhanced forage.

**Conclusions:**

These results suggest that despite their high site fidelity, deer can navigate altered landscapes to track rapid shifts in encounter risk with predators and resource availability. This successful adjustment of movement and behavior following extreme disturbance could help facilitate resilience at broader ecological scales.

**Supplementary Information:**

The online version contains supplementary material available at 10.1186/s40462-024-00488-4.

## Background

Movement is a key trait that allows animal species to adjust to changing landscapes and track resources in dynamic ecosystems, such as fire-prone landscapes [1]. This ability has become increasingly critical in an age of constant anthropogenic global change and extreme environmental disturbances, such as increasingly frequent and severe megafires [2]. In fire-prone ecosystems, megafires, defined as wildfires larger than 100 km^2^ that surpass the size and severity of historical fires, have become increasingly prevalent [3]. Fire has served an important ecological and evolutionary role in many of these ecosystems [4], but historical policy, climate change, and land use change are responsible for the increasing number of unprecedented megafires. Megafires dramatically alter ecosystems at much broader scales than normal fires by rapidly removing resources and triggering rapid conversions in habitat [3]. Though many wild animal species in these fire-prone ecosystems have adaptations to coexist with their historic fire regimes [5, 6], novel megafires may challenge, and even overwhelm, the behaviors and adaptive capacity of individual animals [7]. Behavioral plasticity and movement play an important role in defining an animal’s capacity to adapt and adjust to novel disturbance regimes. Recent work has documented the role movement and behavioral plasticity play in governing the adaptive capacity of species to other forms of global change [8–10]. For large-bodied animals, plasticity in movement and behavior allows individuals to adjust to changes in their local environments [11, 12]. For fire specifically, larger-bodied animals may partition their space-use across recently burned landscapes to take advantage of new resources or avoid risky areas [13].

By rapidly altering landscapes, megafire may impact how some animals are able to navigate and use habitat. Burn severity is a characteristic of fire that defines the loss of below and above ground organic matter [14]. High severity burns, even those outside of megafires, can remove important structural resources from landscapes [15] and even cause direct mortality to animals [16]. Changes in structural cover in these systems may alter interspecies interactions, such as predator-prey dynamics, by altering the success of predator hunting strategies and prey predator-avoidance strategies [17]. Specifically, ambush predators, such as mountain lions (*Puma concolor*), may prefer more unburned areas that maintain cover to successfully ambush prey [18], while more cursorial predators, such as coyote (*Canis latrans*) may prefer more open areas following fire to find prey [19]. High severity fires may remove important food resources (i.e., forbs, grasses, seeds, etc.), potentially influencing herbivorous species populations [20]. Finally, the short and long-term effects of fire on habitat may be directly related to the dominant vegetation type of that habitat. Oak woodland ecosystems, which are a composite mix of grassland, woodland, and shrubland patches, are typically characterized by a moderate or mixed-severity fire regime in which fires burn patchily at low (grass and tree-understory) and high severities (tree-crowns and shrub-crowns) [21]. Recovery times vary across these different vegetation types as well, with grasslands typically recovering faster than oak trees and shrubs. For example, grassland ecosystems typically recover within the first year following fire [22], whereas shrubs and trees may take 5–10 years to fully regenerate following typical woodland fires [23, 24].

Ungulates serve key ecological roles in many fire-prone ecosystems around the world through their herbivory and by serving as a link between different trophic levels. Changes in their movement and behaviors following fire may have important implications for ecosystem-level processes. Fire may influence patterns of ungulate herbivory across landscapes over space and time [25, 26]. Past work has specifically documented a “magnet effect” across several ungulate species, where individuals select moderately burned areas that have improved forage post-fire [27, 28]. Following more severe fire events, recent work suggests that ungulate behavioral plasticity may buffer the short-term impacts of megafire, as ungulates select covered, woodland habitat and expand their home ranges to compensate for a decrease in foraging resources [29]. Kreling et al. (2021) also ask whether these adjustments could become maladaptive to these populations as megafires become more frequent. The seasonality of fire events may also modulate short- and long-term responses of ungulates, with fires potentially increasing the scarcity of rare vegetation resources during the dry seasons or limiting required resources during energetically costly periods of the year (i.e. spring breeding season) [30]. Ungulate browsers, such as Black-tailed mule deer (*Odocoileus hemionus columbianus*) across California, depend on forbs, acorns masts, and tree saplings especially during the dry, non-growing seasons [31], and fires that remove these key resources at these points in time may constrain resources until the next growing season.

Behavioral plasticity mediated by movement likely played an important role in shaping the evolution of ungulate populations in dynamic landscapes where plasticity allowed species to adapt to changes in resource availability caused by fire and other disturbances [32]. Variation in behavior across space permits animals to respond accordingly to dynamic landscapes [33, 34]. Behavioral plasticity, therefore, likely continues to play a significant role in influencing the resilience of ungulate species to major wildfire events and changes to local fire regimes. Unlike other large ungulates, behavioral plasticity of mule deer migratory movement specifically has been found to be non-plastic or rigid [35]. Alternatively, previous work has also established that mule deer are capable of efficiently navigating burned landscapes to simultaneously minimize predation risk from a variety of predators whilst identifying and using areas with forage [36]. Therefore, deer may not change the location of their range, even if it is entirely burned in large-scale events such as megafires, but they may alter their space usage within these large burn extents. Understanding the conditions and thresholds under which behavioral plasticity is adopted as an adaptive strategy may be key in tailoring management for this species following major environmental disturbances. Megafires may overwhelm this capacity, and it may take much longer for species to recover to pre-fire conditions due to the dramatic changes imposed on the landscape. It’s important to understand how ungulates adjust their behavior in burned areas over time. This insight is crucial for assessing adaptability to changing fire regimes and guiding management strategies.

In this study, we examined the long-term consequences of megafire on an ecologically and economically important Californian ungulate, the black-tailed mule deer. As a direct follow-up to a study on the initial effects of the 2018 Mendocino Complex Fire on deer behavior [29], we investigate how short-term movement responses of deer to megafire vary over the year following fire. Due to the scale and severity of this megafire, we hypothesized that (1) observed changes in deer behavior (increased home range size and habitat preferences) would persist until the end of the study period. Secondly, we hypothesized that (2) deer would preferentially select habitat that burned at low severity immediately following the fire (“Recently Burned”) to avoid exposure to predators and select areas more likely to have forage remaining. This, however, may not be the case if the activity of mountain lions, the predominant predator of deer in this system, decreases in exposed areas that lack the cover conducive for ambush hunting. In line with the magnet effect, (3) we predicted black-tailed deer would select areas that burned at moderate severities the following growing season (“First Spring”) due to the increased nutritional value of forage in these areas. We hypothesized that the behavioral plasticity of deer would allow them to adjust their movement to minimize risk and maximize access to resources. Specifically, we hypothesized that (4) black-tailed deer would be more likely to travel through severely burned areas to avoid exposure to potential predators, and to rest in low severity burned areas where perceived risk may be lower. Similar to deer habitat selection, changes in deer behavioral modes are likely dependent on whether the activity of their predominant predator, mountain lion, changes in these severely burned areas as well where ambush hunting may be less successful. Large, high severity patches of this fire removed extensive shrub cover and top-killed patches of oak woodland throughout the study site. We predicted these large-scale, structural changes would lead to long-lasting behavioral adjustments in both habitat selection and behavioral modes that would be consistent throughout the study period.

## Methods

### Study site and fire history

**Fig. 1:**
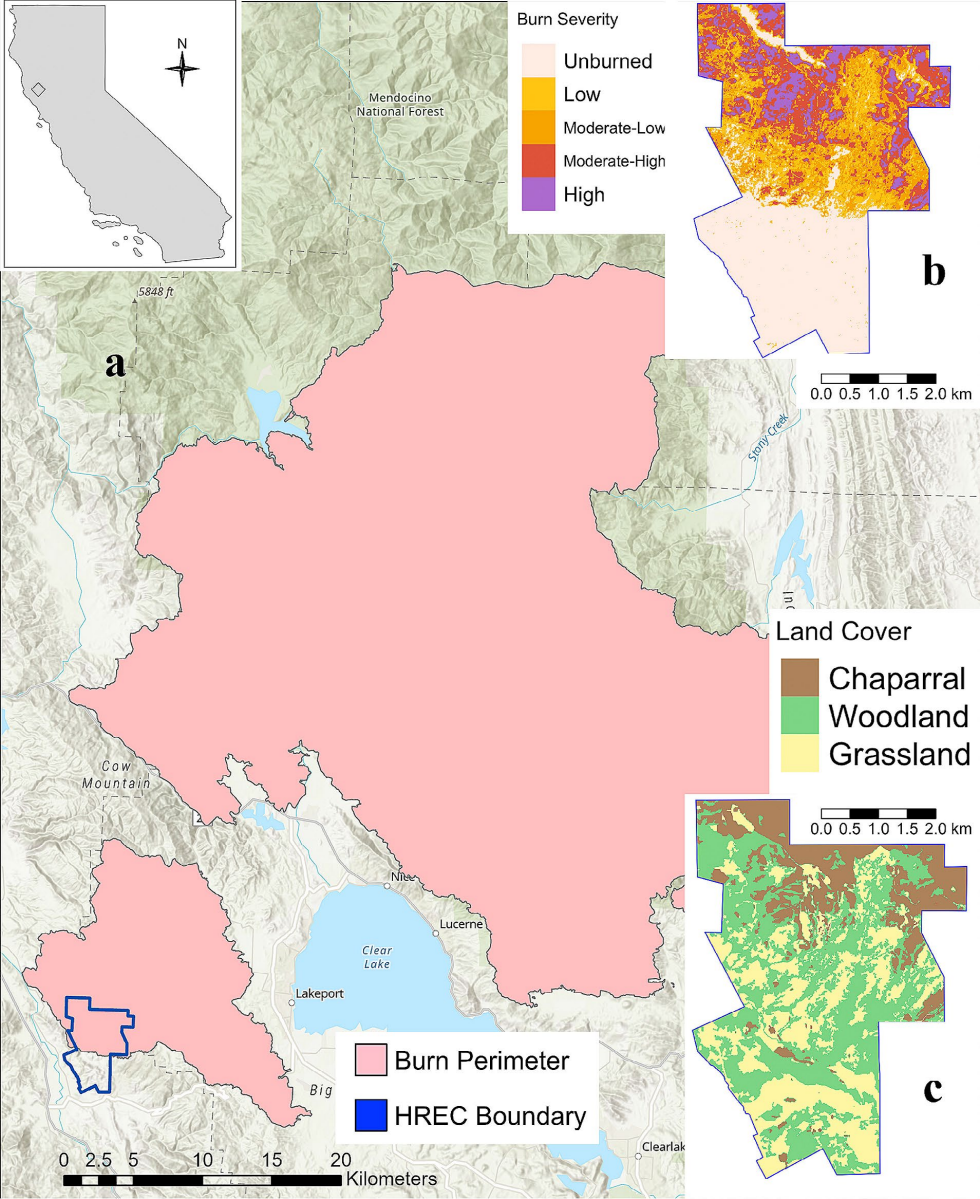
Maps of the 2018 Mendocino Complex Fire and the study site, the U.C. Hopland Research and Extension Center (HREC) (39°00′ N, 123°04’ W). Map “**a**” displays the total burn perimeter of the Mendocino Complex Fire. This fire burned into HREC on July 27, 2018. Map “**b**” displays the severity of the fire across the HREC property boundary. Sentinel-2 satellite imagery was acquired via Google Earth Engine to calculate fire severity. Fire severity was quantified as the Differenced Normalized Burn Ratio (dNBR). For visualization purposes, dNBR values were binned into categorical values based on those established by US Geological Survey as follows (Unburned = 0–99, Low = 99–269, Moderate-Low = 269–439, Moderate-High = 439–659, High = 659+). Map “**c**” displays the compositional makeup of dominant vegetation types across HREC. In this map, yellow denotes grassland, green denotes woodland, and brown denotes chaparral shrubland

We conducted this study at the Hopland Research and Extension Center (HREC hereafter) in Mendocino County in northern California (39°00′ N, 123°04’ W) (Fig. 1). HREC is composed of a diverse set of vegetation types including chaparral shrublands (*Adenostoma fasciculatum*), oak woodland savannah (*Quercus kelloggii*, *Quercus douglassi*, and *Quercus lobata*), and a mix of introduced and native, open grassland. HREC is characterized by a Mediterranean climate with cool, wet winters and warm, dry summers. HREC also operates as a working rangeland landscape, containing a sheep farming facility and several agricultural plots throughout the property [37]. Deer hunting occurs seasonally on site generally from August-September. A maximum of 120 hunters are allowed on site annually (20 hunters per day) and an average of 23.4 deer are harvested annually [38]. No hunting was permitted during 2018 because of megafire. Mountain lions are the predominant predator of mule deer on site, but coyote and black bear (*Ursus americanus*) also occur on site and have been observed to prey on deer and their fawns periodically [39].

On July 27, 2018, the River Fire (southern half of the Mendocino Complex Fire), swept through the northern half of HREC, burning approximately 13.76 km^2^ (65%) of the property. The 2018 Mendocino complex fire burned 1,858 km^2^ total and is currently the third largest wildfire in California’s recorded history (CALFIRE-FRAP, 2022). Fires in this region typically burn relatively frequently every 5–15 years at relatively low severities in the more open woodland and grassland habitats and more infrequently, but more severely, in the dense shrubland chaparral habitats every 30–60 + years [40, 41]. The River Fire burned a much larger contiguous area and much more severely than recent fires within HREC. Atypical of fires in woodland fire regimes, several oak trees (*Q. kelloggii*, *Q. douglassi*, and *Q. lobata*), whose acorn masting normally provides a key food resource for local deer populations, were top-killed in certain high severity patches of the this fire. Acorn masting remains highly variable across different oak species, as well as between individual trees, but mature trees typically mast every 2–3 years dependent on climatic factors and interspecies interactions [42].

### Monitoring black-tailed deer movement and home range estimation

We deployed GPS-collars (Vertex Plus and Lotek Iridum Track M) across 28 female deer between July 2017 and July 2019. These data provided the basis for a natural experiment to observe the effects of megafire on deer movement and behavior. We programmed all collars to record GPS locations once per hour. Deer were captured using Clover traps and were manually restrained to place collars on, without the use of chemical immobilizers (permit #P1680002). We monitored deer remotely post-capture for 1 week to ensure collared deer did not experience capture myopathy and that the distance they traveled within a day was typical of a healthy individual.

To observe how deer movement and behavior changed over time following the megafire, we subset the collected GPS data to only include deer that had GPS points that overlapped the fire perimeter of the Mendocino Complex Fire, excluding seven individual deer. We then subset the collected GPS points temporally into three two-month-long time periods: just after the fire (August 1st – October 1st 2018), the first spring green up following the fire (March 1st – May 1st 2019), and one full year post-fire (August 1st – October 1st 2019) (Additional File 1 - Table S1). We included two additional pre-fire time periods to compare deer home range size before and after the fire and to examine any seasonal differences in home range size that may impact our results. These additional pre-fire time periods included: two separate spring seasons before the fire (March 1st – May 1st 2017 and March 1st – May 1st 2018) and just before the fire (May 25th – July 25th 2018). We collected > 1,000 GPS fixes for most deer within each time period (29 of 38 deer-periods) (Additional File 1 – Table S2). The “Prefire” time period occurs at the intersection of our annual collaring efforts when many collars from the previous year are programmatically dropped off and collars for the coming year are deployed. This resulted in us collecting fewer GPS locations from individual deer during this time period. To facilitate analyses that included this key time period, we therefore included deer-periods that had a minimum of 500 recorded GPS locations, excluding four individual deer-periods. This resulted in 21 unique individuals collared across these five time periods. Thirteen of these animals maintained their collars across two or more time periods, resulting in 38 period-specific deer home ranges (Additional File 1 – Table S2). We removed 10 erroneous, outlier GPS locations that were greater than 2 km from their consecutive points for these deer between hour fixes.

For each deer, and within each time period (deer-period), we used the two months of collected GPS data to estimate individual home range sizes. We used 95% Kernel Utilization Densities (KUD) in the “adehabitatHR” (v.0.4.19) package in “R” (v.4.1.1) to create these home ranges [43–45]. To assess whether deer home range sizes continue to change following the megafire, we used paired Welch’s unequal variance t-test to compare deer home range sizes 1) just after fire (“Recently Burned”), 2) the first spring following fire (“First Spring”), 3) one full year post-fire (“1 Year Post-Fire”), 4) the spring seasons before fire (“Pre-spring”), and 5) just before the fire burned (“Pre-fire”). To assess the robustness of this analysis to the smaller sample size of under sampled time periods (i.e. “Prefire”), we randomly sampled 500 GPS locations from each deer-period and repeated this analysis to compare changes in home range size with the rarefied data as well.

### Environmental covariates

We compiled fire and other environmental covariates alongside deer movement data to evaluate black-tailed deer movement responses to the megafire over time. We expected that fire severity, predator encounter probability, vegetation type, distance to water, and time since burning would be strong predictors of both deer habitat selection and deer movement during each post-fire time period. We originally planned to include NDVI (Normalized Difference Vegetation Index) as a measure of forage availability across the landscape, but we found measures of NDVI were highly correlated with measures of fire severity, our primary covariate of interest. Therefore, we included fire severity and excluded NDVI. To quantify fire severity on the landscape [46], we calculated the differenced Normalized Burn Ratio (NBR) collected via Sentinel-2 [47] satellite imagery (10 m resolution) and processed in Google Earth Engine [48] from both before (July 25th, 2018) and after (August 25th, 2018) the fire. NBR was calculated using the following equations (14):

*∆NBR = NBR*_*prefire*_*- NBR*_*postfire*_.

*NBR = Near-infrared (NIR) – shortwave infrared (SWIR) / Near-infrared (NIR) + shortwave infrared (SWIR)*.

We also included a quadratic term for fire severity to examine whether deer may preferentially select for moderately burned areas that, according to the magnet effect, may eventually have more nutritious forage after vegetation regrowth.

To account for mountain lion encounter probability across the landscape for this study, we included a high-resolution mountain lion habitat suitability map produced for the entire State by Dellinger et al. 2020 in our analyses [49]. This habitat suitability modeling effort used a suite of biotic and abiotic variables, including terrain ruggedness, canopy cover, and a rough categorical estimate of deer density. Though predator occurrence is not necessarily associated with predation risk [50], and we do not have data of observed mountain lion kills at the site to create a study-specific risk map, we used this habitat suitability map to serve as a proxy of perceived risk [51] for deer across our study site.

We classified the study site into three broad land cover categories: woodland, shrubland (chaparral), and grassland. To do this, we hand digitized vegetation layers using high-resolution (< 1 m) aerial imagery from the National Agriculture Imagery Program (2014–2015). In 2015, we ground truthed these digitizations by checking 50 randomly generated points across the study site to validate classifications (results were 98% accurate). Our primary interest was to compare the strength of selection and avoidance of broad vegetation types by deer following fire. Therefore, we chose to represent vegetation types as three dominant land cover categories as opposed to continuous covariates. Previous work in this region found that deer prefer woodland habitat following fire [29], so we chose to use woodland as the reference category within our model to compare against the other vegetation types. To calculate the distance between collected GPS points and potential water sources, we obtained stream vector data from the National Hydrography Dataset and used the “sf” (1.0.2) package in R to calculate the distance between collected GPS locations and seasonal streambeds throughout the study site.

We checked the (Variance Inflation Factor) score of covariates to ensure there was no underlying collinearity between modeled covariates (VIF < 3) and qualitatively inspected plotted covariates as well (Additional File 1: Figure S1; Additional File 1: Figure S2).

### Resource selection functions

We used Resource Selection Functions (RSFs) to assess black-tailed deer habitat selection across each post-fire time period. Previous work illustrates that point (RSFs) and path (SSFs) selection functions work similarly well in defining habitat selection [52], but given the small home range sizes for most deer in our study and their proportionally large step-lengths, we chose to employ RSFs specifically as most of their defined home range should be “available” to use at any given time step. We collected > 1,000 GPS locations for all deer-periods used in this analysis (26 of 38 deer-periods, 16 unique individuals) (Additional File 1: Table S2). For these RSFs, we used the home ranges defined by the 95% Kernel Utilization Densities (KUDs) [43–45]. We modeled habitat selection for all time periods combined to improve interpretability of model results. We also included an interaction term between time period and severity (Severity*BurnLag). For each deer-period home range, we randomly generated four-times as many “available” points from within each deer’s estimated KUD home range (mean available point density across all modeled deer = 8,174.72 available points/km^2^) [29]. Available points were stratified by time period so that the number of available points had the same ratio across time periods as the true use points. We compared the environmental characteristics of “used” and “available” GPS points using a mixed effects logistic regression via the “lme4” (v.1.1.27.1) package in R [45, 53].

We used an *a priori* hypothesis-driven approach to select a model to describe deer habitat selection, that included fire severity and its quadratic term (to account for nonlinear effects), encounter probability with mountain lions, vegetation type (chaparral, woodland, or grassland), distance to water, time since burn, and an interaction between severity and time since burn as covariate predictors. We used woodland as the reference vegetation category within these RSFs. We randomly sampled “time since burn” for each available point as a randomly selected date from within its respective time period. Prior to modeling, we standardized each of the included covariates (mean = 0, standard deviation = 1). We included a random intercept of “Deer ID” within our RSF to account for individual differences in behavior and resource availability for each deer (individual deer retained their same “Deer ID” across time periods).

To assess goodness of fit of the RSF model, we used the “performance” (v.0.7.3) package in R [54] to calculate marginal and conditional *R*^*2*^ values for the model and visually inspect overall model fitting.

### Hidden markov movement models

While examining habitat selection provides an important opportunity to uncover *where* animals tend to spend time across landscapes, it is equally important to understand *how* animals use the time they spend in the areas they are selecting or avoiding as mediated by behavior.

By defining certain movement parameters (i.e. turning angle and step-length), we can use hidden Markov movement models (HMMs hereafter) to predict behavioral states of animals at individual GPS-fixes and compare how the distribution of these states may change in response to environmental covariates across a landscape, such as fire [55]. These behavioral states represent types of responses to an animal’s environment such as “foraging”, “traveling”, or “resting” [56].

To assess how deer behavioral decisions were impacted by megafire, we fit a HMM across the combined, three post-burn time periods within our study (26 deer-periods, 16 individuals) using the “moveHMM” (v.1.8) package within R [57]. We modeled two behavioral states (state 1 = resting, state 2 = traveling) to increase model interpretability and to specifically observe whether deer traveling and resting behavior changes across landscape variables to avoid perceived risks following fire. We calculated step lengths (via von Mises distributions) and turning angles (via gamma distribution) to characterize the two behavioral states. We randomly generated 25 different pairs of starting values from a range of plausible values as defined by the range of each calculated movement parameter (step-length and turning angle) (Additional File 1: Table S3). We ran each of the 25 randomly generated step-length and turning-angle pairs in a model without covariates and compared the negative-log likelihood of each model. We checked that each model had similar maximum log-likelihood values and we selected the best fitting pair of movement parameters based on maximum likelihood [58].

Using these starting values, we fit a single hidden Markov model with a set of *a priori* selected covariates (Severity + Mountain Lion Encounter Probability + Distance to Water + Time Since Burn + Vegetation Cover + Severity*Time Since Burn) to estimate how the probability of being in a certain behavioral state (i.e. resting vs. traveling) changed as a function of these environmental factors. We then used the “stationary” function of the “moveHMM” package to estimate the probability of each GPS point being in a given behavioral state and used these to create activity budgets by summing the estimated probabilities for being in each state at each recorded GPS point [59]. We used a Chi-squared test to assess whether the proportions of the two behavioral states were significantly different across time periods.

We assessed goodness of fit for the HMM using pseudo-residuals drawn from the fit model. Pseudo-residuals of the step length parameter should be normally distributed given good model fit [60, 61]. Therefore, we visually inspected step length pseudo residuals and used a Shapiro-Wilk normality test using a random subset of pseudo residual values (*n* = 1000).

## Results

### Home range comparison across seasons

**Fig. 2:**
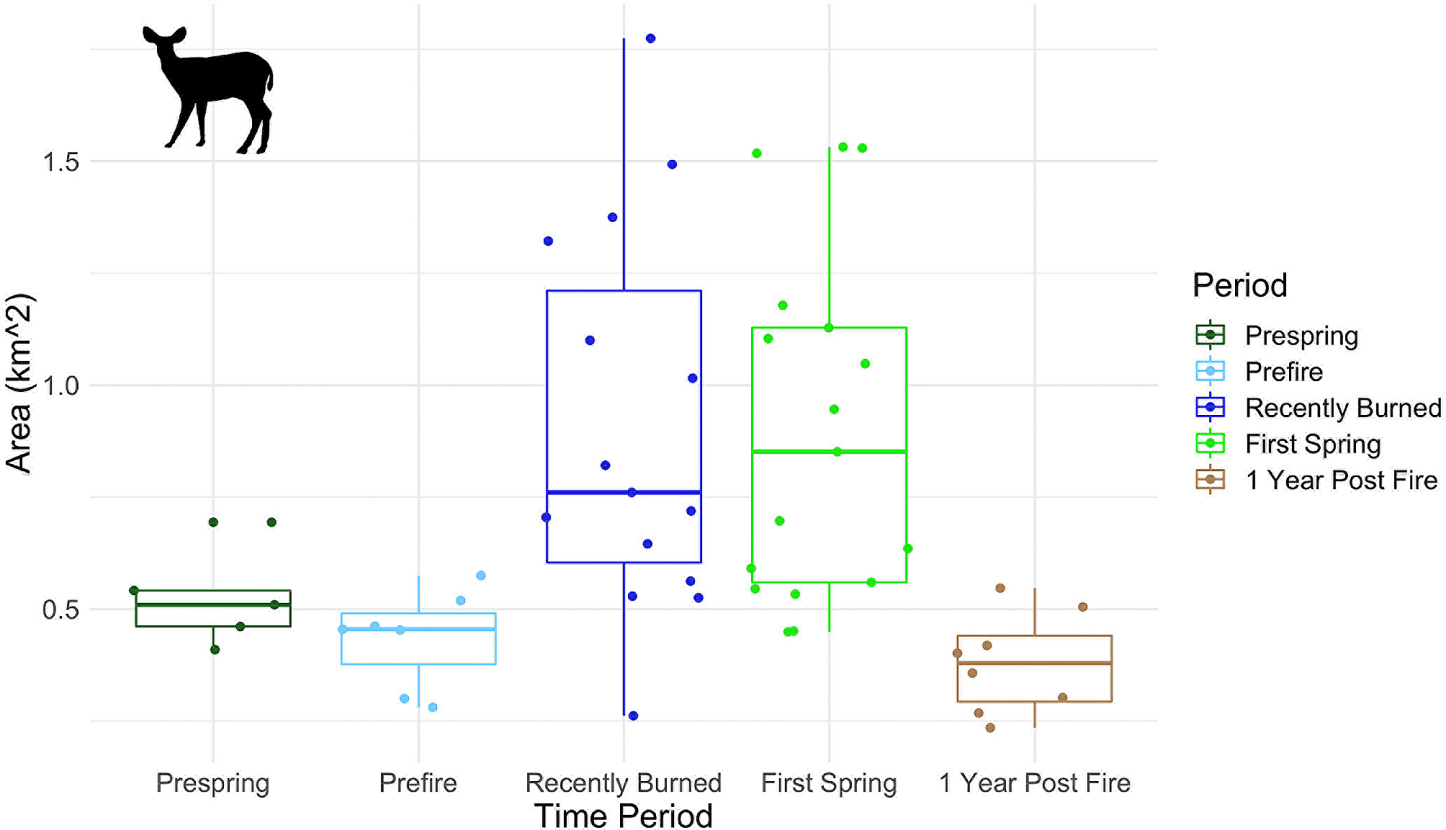
Home range size of black-tailed deer (*Odocoileus hemionus columbianus*) across five time periods both before and after the 2018 Mendocino Complex Fire in Hopland, California, USA. The Mendocino Complex Fire burned July 27th, 2018. These study periods include: 2017 Spring and 2018 Spring before the fire (“Prespring”), the summer season just before the fire burned (“Prefire”), directly following the fire (“Recently Burned”), the first spring following the fire (“First Spring”), and 1 full year post fire (“1 Year Post Fire”) (*from left to right*)

We found that the average deer home range size across all time periods was 0.75 km^2^ (sd ± 0.42). Deer home range sizes were largest in the two time periods directly following the fire (“Recently Burned” and “First Spring”) and were smallest in the two pre-fire time periods (“Prespring” and “Prefire”) as well as “1 Year Post Fire”. The average home range size was 0.95 km^2^ (sd ± 0.46) during the “Recently Burned” period and 1.08 km^2^ (sd ± 0.35) during the “First Spring” period. During the “1 Year Post Fire” time period the average home range size was 0.38 km^2^ (sd ± 0.13). Finally, during the pre-fire time periods, the average deer home range size was 0.43 km^2^ (sd ± 0.11) for the “Prespring” time period and 0.52 km^2^ (sd ± 0.11) during the “Prefire” time period. (Additional File 1: Table S1; Fig. 2). We found no significant difference between deer home range sizes during the “Recently Burned” and “First Spring Periods (t = -0.72, df = 14.71, p-value = 0.48). We did find a significant difference (p-value < 0.05) in deer home range size between the “Recently Burned” and “1 Year Post Fire” periods (t = 3.52, df = 9.82, p-value < 0.01), as well as between the “First Spring” and “1 Year Post Fire” periods (t = 5.98, df = 13.95, p-value < 0.01). We found no significant differences between the home range sizes of the two pre-fire time periods, “Prespring” and “Prefire” (t = 1.26, df = 6.11, p-value = 0.25). We also found no significant difference in home range size between the “1 Year Post Fire” and “Prespring” periods (t = 1.396, df = 8.787, p-value = 0.20), as well as between the “1 Year Post Fire” and “Prefire” periods (t = -0.83, df = 9.80, p-value = 0.42) (Table S4).

We found identical results in our additional analysis using a subset of 500 randomly selected points from each deer-period suggesting that our analysis is robust to differences in sample sizes across time periods (Additional File: Table S5; Additional File: Figure S3).

### Resource selection functions

**Fig. 3:**
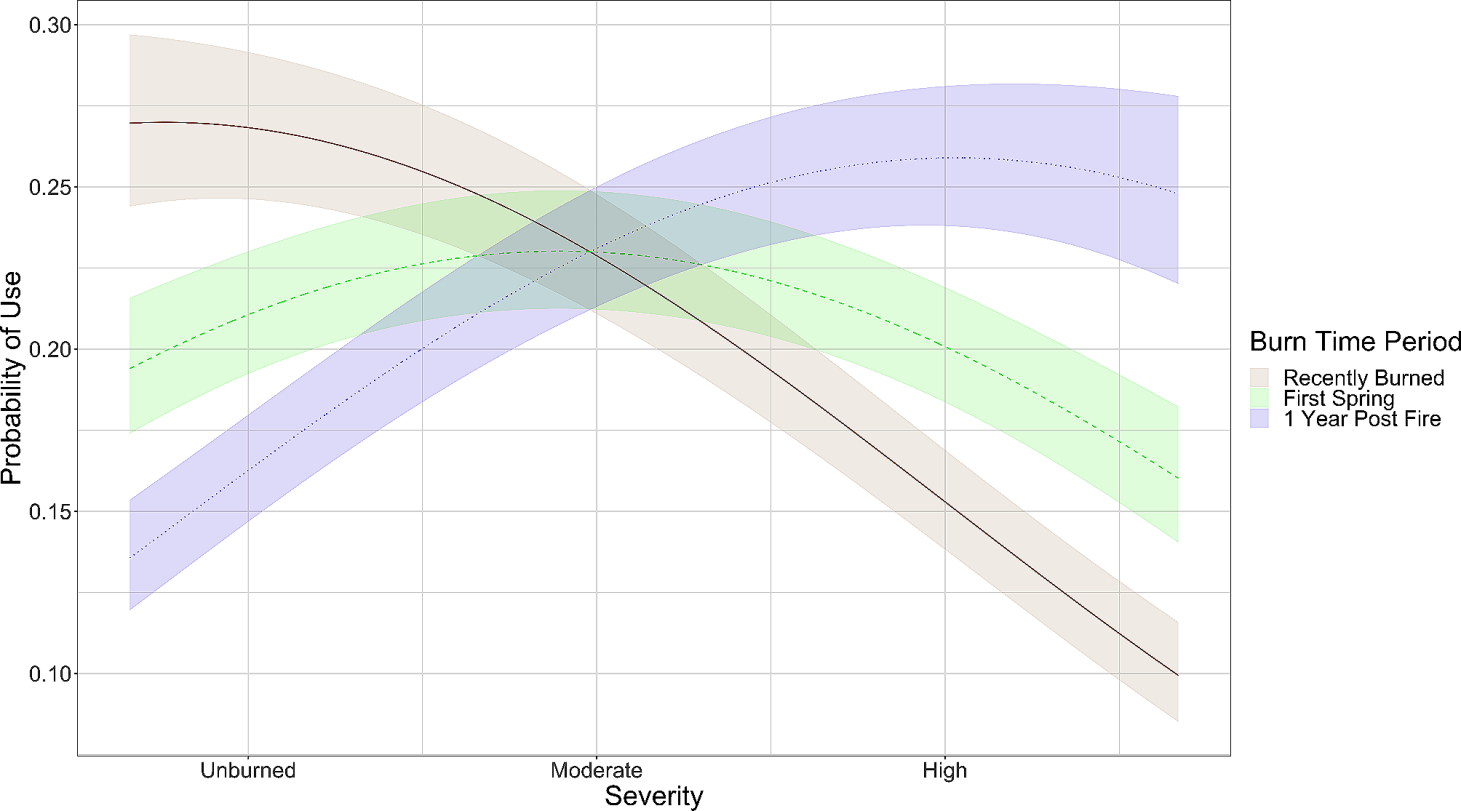
Plotted response curves of deer (*Odocoileus hemionus columbianus*) habitat selection in response to fire severity and time since fire, as predicted from a resource selection function following the 2018 Mendocino Complex Fire at the Hopland Research and Extension Center, CA, USA. To visualize the interaction, we used the midpoint date of each time period to represent a categorical “Time Since Burn” variable in the plot

Overall, deer avoided areas that burned at high severity, but this response was nonlinear and probability of use was highest at intermediate severities (Table 1). However, we also found that deer habitat selection of fire burned areas changed over time as an interaction with time since burn. During the “Recently Burned” time period, deer were more likely to avoid high severity areas (Additional File 1: Figure S4). Conversely, deer selected higher severity burned areas during the final “1 Year Post Fire” period (Fig. 3). Deer preferred woodland habitat over grassland and chaparral following the fire (Additional File 1: Figure S4). Deer also avoided areas of high mountain lion encounter probability (mean = -0.09 [-0.08, -0.10]) (Table 1).

**Table 1:** Listed output estimates for each covariate of the resource selection function for black-tailed deer (*O. Hemionus columbianus*) following the 2018 Mendocino Complex Fire at the Hopland Research and Extension Center in Mendocino County, CA, USA. Beta-coefficients, standard errors, and p-values are listed for each covariate included in the model. For categorical vegetation types, “woodland” was used as the categorical variable. * indicates statistically significant predicter of habitat selection within the model (p-value < 0.05)

Covariate	β-Coefficient	95% CI	*p*-value
Intercept	-1.21	[-1.15, -1.26]	< 0.001*
Severity	-0.02	[-0.02, -0.03]	0.001*
Severity Squared	-0.04	[-0.03, -0.04]	< 0.001*
Mountain Lion Encounter Probability	-0.09	[-0.08, -0.10]	< 0.001*
Chaparral	-0.41	[-0.39, -0.42]	< 0.001*
Grassland	-0.16	[-0.15, -0.18]	< 0.001*
Time Since Burn	0.02	[0.03, 0.01]	0.01*
Distance to Water	0.01	[0.01, − 0.01]	0.90
Severity * Time Since Burn	0.16	[0.17, 0.15]	< 0.001*
Observations	170,708		
Conditional R^2^	0.030		
Marginal R^2^	0.018		

### Hidden markov model results

**Fig. 4:**
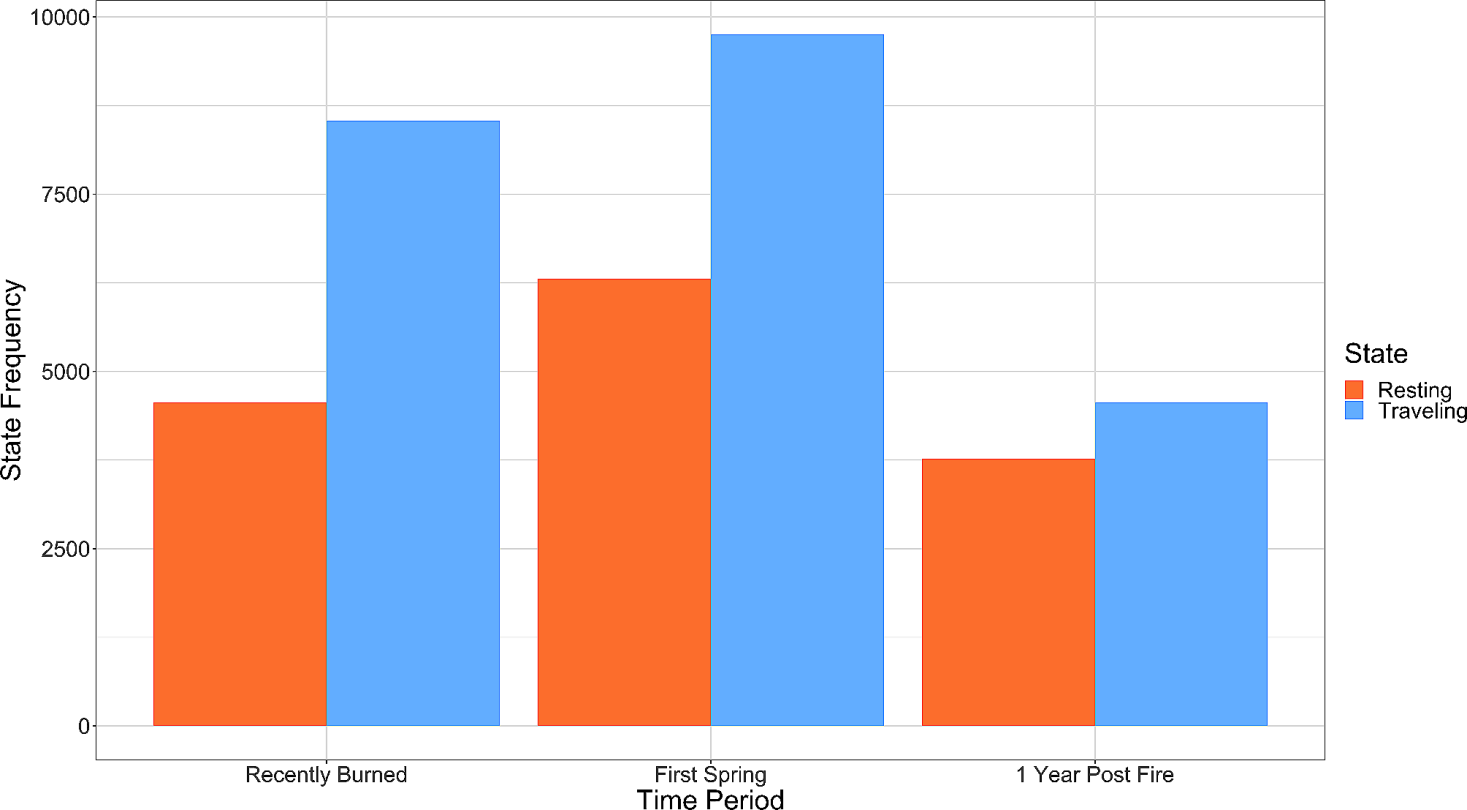
Behavioral state proportions for black-tailed deer (*Odocoileus hemionus columbianus*) at the Hopland Research and Extension Center in Mendocino County, California. Behavioral states for deer tracks were estimated for each post-fire time period by the hidden Markov model. State frequencies represented the summed probabilities of each GPS point being in a specific behavioral state

We found that the 25 iterations of our null model converged on very similar scores of maximum likelihoods (mean = 266750.40; sd = 956.38). Our best fit hidden Markov model estimated two deer behavioral states: a “resting” state with shorter step-lengths and wider turn angles and a “traveling” behavioral state with longer step-lengths and near 0 turning angles (Additional File 1: Figure S5; Additional File 1: Figure S6). We found a significant difference in the composition of behavioral states between all time periods, with deer spending a greater proportion of time traveling than resting immediately following fire and during the first spring (χ^2^ = 232.97, df = 2, p-value < 0.001) (Fig. 4; Additional File 1: Table S6) compared to the proportion of time spent in each state during the “1 Year Post Fire” time period.

**Fig. 5:**
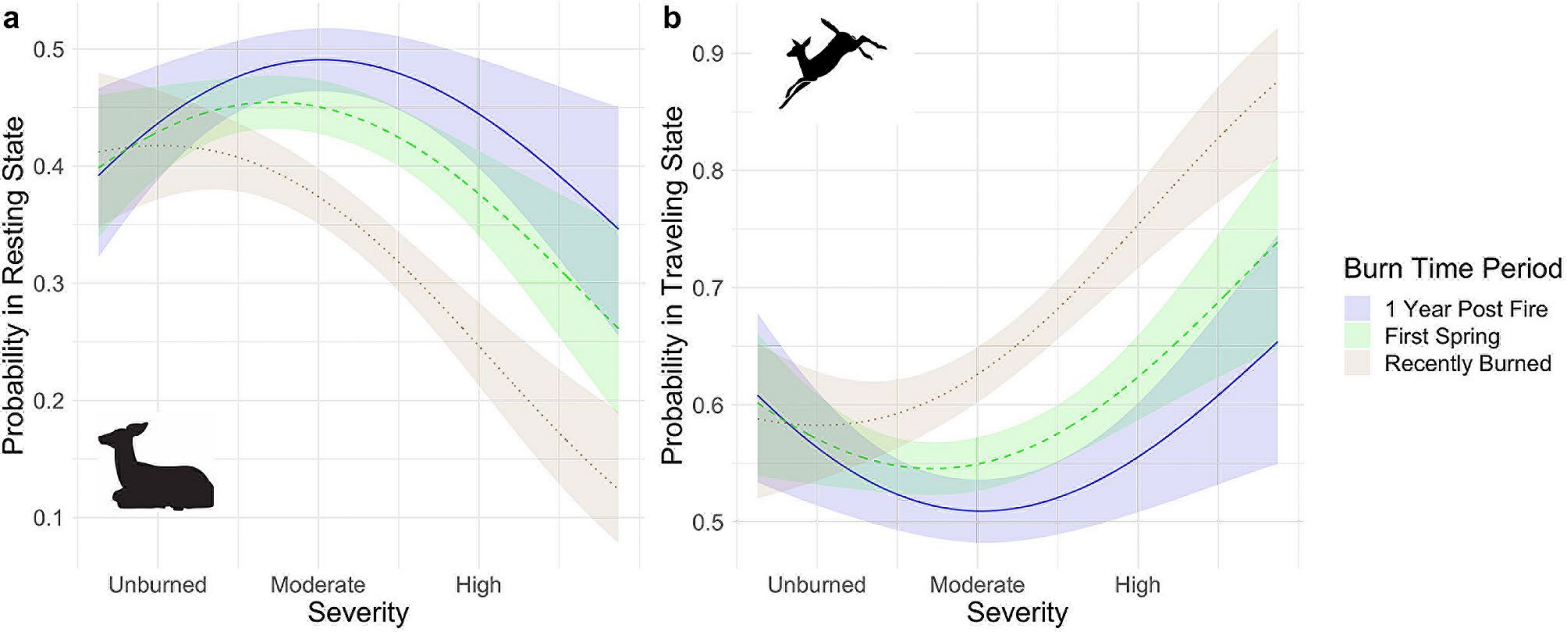
Behavioral state probabilities of black-tailed deer (*Odocoileus hemionus columbianus*) as a function of fire severity and time periods following the 2018 Mendocino Complex Fire at the Hopland Research and Extension Center in Mendocino County, California. Plot **a**) displays the probability of deer being in the “resting” behavioral state as a function of fire severity across the three time periods (“Recently Burned”, “First Spring” and “1 Year Post Fire”). Plot **b**) displays the probability of deer being in the traveling as a function of across the three same time periods. Shaded areas represent 95% confidence intervals. *Note that y-axis scaling of plots a and b are different

We found that deer behavioral states changed as a function of fire severity. Deer were most likely to be in the “resting” behavioral state in unburned and moderately burned areas across all time periods (Fig. 5a). At high severities, deer were more likely to be in the “traveling” behavioral state. The probability of deer being in the “traveling” behavioral state at high severities was significantly higher during the “Recently Burned” time period than in the “First Spring” and “1 Year Post Fire” time periods (Fig. 5b).

Pseudo residuals drawn from the HMM suggested good model fit for the deer track data. Overall, plotted pseudo-residuals of deer step-lengths appeared normally distributed (Additional File 1: Figure S7). We failed to reject the null hypothesis of the Shapiro-Wilks significance test (W = 0.99, p-value = 0.61), suggesting pseudo-residuals were drawn from a normal distribution.

## Discussion

In this study, we utilized deer movement data collected opportunistically before, during and after a megafire to examine how deer behavior and space use changes following severe environmental disturbances. We found evidence that refuted our initial hypotheses that deer home range size and habitat selection would remain constant throughout the study period due to the severity and size of the megafire. Instead, estimated deer home range size and habitat selection was shown to change significantly throughout the course of the study. We found evidence supporting our hypotheses that deer preferred low severity burned areas immediately following the megafire and preferred moderate-high severity burned areas later in the study. Finally, we found evidence supporting our hypothesis that deer have some behavioral flexibility in adjusting their movement behaviors (traveling vs. resting) across varying burn severities. The distribution of these behavioral modes across low and high severity burned areas also changed over time. Our results demonstrate the mechanisms in which movement and behavior underpin the capacity of black-tailed deer to effectively adjust to a quickly shifting landscape follow megafire.

Contrary to our original hypotheses, we found that black-tailed deer habitat selection and the composition of movement-inferred behavioral states changed as a function of fire severity and time. As Kreling et al., 2021 found, ungulate home ranges were larger directly following the megafire, but we found that this effect does not persist over time. Deer home range size was significantly higher during the first two time periods following megafire (“Recently Burned” and “First Spring”) compared to the pre-fire time periods (“Prespring” and “Prefire”) and “1 Year Post-Fire”. The scale of the change in home range size observed in this study exceeds what has been previously observed in other studies caused by normal inter-season variation [36, 62], suggesting megafire had a significant effect on deer home range size and space usage. These results corroborate the conclusions drawn from Calhoun et al. 2023, which found that the intensity at which black-tailed deer used recently burned areas decreased during the year of this megafire, but returned to pre-fire conditions one-year following the megafire [63]. Deer space use likely becomes more diffuse as their home ranges expand immediately following fire.

Directly following the megafire, deer strongly avoided areas that burned at high severity, but this effect waned in the initial spring months following fire and inverted by the “1-Year Post-Fire” time period, with deer instead selecting for habitat that burned at higher severities. Similarly, deer were more likely to move than to rest in high severity areas immediately following megafire, but this effect diminished over the course of the year. Our results suggest that the behavioral adjustments made by individuals may be effective coping mechanisms for the potential consequences of megafire. This observed behavioral plasticity may also allow deer to eventually take advantage of the resulting resources that become available over time.

Fire severity was a significant predictor of deer habitat selection, but we found that the direction of selection (against high severity areas vs. towards high severity areas) changed as an interaction with the amount of time that had passed since the fire burned. As observed in previous studies [29], black-tailed deer avoided high severity burned areas in the immediate aftermath following the fire. These observed behaviors may be used to avoid exposure to predators in open areas, but the exact mechanism requires further research. While deer may perceive greater predation risk in these exposed areas, mountain lions, their primary predators, may be paradoxically less likely to use recently burned, uncovered areas that limit ambush success [17, 18]. Alternatively, recent work in this same system has shown that coyote, an alternative predator, increases their occurrence and activity in recently burned areas [63], potentially dissuading deer from using these areas as well. This may be especially pertinent for does with fawns or yearlings that are vulnerable to predation by coyote [39]. Future work that compares the influence of perceived and actual predation risk across these potential predators could better elucidate the mechanism underlying these observed responses.

In agreement with our initial hypothesis, we found that deer began to select areas that burned at moderate severities during the first spring green-up. However, we did not anticipate that deer would select high severity burned areas during the final time period of the study (“1-Year Post-Fire”). We expected that the high severity burned areas would be depleted of resources for the duration of our study, but our results suggest that once the vegetation in these severely burned areas is able to recover, these areas may attract herbivorous species [64]. This is at least partially supported by a brief, qualitative look at the relationship between forage quality, as represented by EVI (Enhanced Vegetation Index), and fire severity in the latter time periods of the study (Additional File 1: Figure S8) in which forage quality is higher at moderate severities during “First Spring” and increases at high severities during “1 Year Post Fire”. Both ‘First Spring’ and ‘1 Year Post-Fire’ time periods support the ‘magnet effect,’ observed in other studies, where ungulates prefer recently burned areas with enhanced forage [65, 66]. This emphasizes fire’s significant role in ungulate ecosystems, despite broader changes in fire regimes and climate.

Changes in deer behavior over time following fire are likely mediated by how and when vegetation recovers. Though we anticipated that changes in habitat selection would persist longer in severely burned woodland and shrubland areas that take longer to regenerate relative to grasslands [23, 24], our results suggest that these fire-adapted, early-successional plant species can still provide new food resources over much quicker time scales. Alternatively, fire-following forbs and new tree shoots may provide a source of new foraging opportunities for herbivores, particularly for browsing ungulates [67].

The proportion of estimated deer behavioral states changed over the course of the study and the probability of deer being in certain behavioral states (traveling vs. resting) varied significantly with fire severity. However, contrary to our initial hypotheses, we found the probability of deer being in a certain behavioral state as function of severity was not constant across time periods, but instead changed across time. Initially following megafire, we found that deer were more likely to travel through high severity burn areas and spent more time resting in low severity areas. This strategy may allow deer to avoid spending too much time in exposed areas, and to spend more time in the limited areas that contain food and shelter [13]. Spending less time traveling in exposed areas during the late summer may be critical for thermoregulation, specifically to avoid heat stress, and may be especially important for does with fawns [68, 69]. Immediately following megafire, deer spent significantly more time in the “traveling” state than the “resting” state, but this effect waned over time. This, as both a result of and in combination with an overall decrease in resource availability, may potentially result in the initial decreased body condition of ungulates following megafire observed in the study area [29]. Despite this, our findings suggest that black-tailed deer may find benefits in moderate-high severity burned areas over an extended period following megafire that may outweigh these initial costs, but additional research is required to examine how deer body condition changes over time.

Our results suggest that black-tailed deer in this study area have great capacity for short-term behavioral plasticity to allow quick adjustments of their behavior patterns in response to disturbance and vegetation recovery. This mirrors similar findings in nearby deer populations that were found to adjust to seasonal changes in food availability by adjusting their habitat selection in the summer and winter months [70] and may suggest a broader evolutionary adaptation to these types of extreme fire regimes [71]. While the overall extent of this megafire may represent an extreme disturbance event, aspects of this disturbance still resemble key evolutionary pressures to which these animals have adapted to. Behavioral adaptions to dynamic, fire prone landscapes may facilitate deer decisions to maintain high site fidelity in anticipation of the eventual regrowth of increased vegetation resources in burned areas as time passes [72]. While previous work has shown that species traits and fire characteristics play an important part in creating mammalian resilience to fire disturbances [73], this study highlights the key role of behavior underlying these observed effects.

Our results highlight the resilience of black-tailed deer and oak woodland ecosystems overall to megafire via adaptations both have evolved within fire-prone ecosystems. The strategies several plant and animal species have acquired by evolving in fire-prone ecosystems may also be employed when responding to megafire. For large-bodied and vagile animals in particular, movement and behavioral strategies used to adjust within landscapes that burn regularly likely play a significant role in facilitating resilience to changes in resource availability caused by megafire as well [74]. Species in more dynamic landscapes have likely developed some degree of behavioral plasticity to deal with the unpredictability of resources more effectively [75, 76]. This behavioral plasticity may also buffer these species from other types of climatic disturbances [77–79], and should be incorporated in future studies that examine the predicted impacts of climate change on species conservation. This study also conveys how movement and behavior are the primary mechanisms by which resilience to megafire may be facilitated as observed in previous work [63]. The success of behavioral plasticity in buffering the effects of megafires likely depends on the degree to which these events depart from the historical fire regimes these species have adapted to [7]. We therefore require more work that can better define the thresholds of extent, severity, and frequency for specific megafires that may overwhelm the behavioral plasticity of ungulates and other fire adapted species.

We found that deer selected for woodland habitat relative to the other vegetation types and strongly avoided shrubland habitat. Deer likely avoided these open shrubland areas to avoid conspicuous encounters with predators [80] or to avoid exposure to temperature extremes in the late summer. Future work that compares these two potential mechanisms using concurrent movement data from ungulates and their predators to asses changes in predation [17], or physiological data related to the thermoregulatory cost of heat exposure could help fill this remaining gap. The limited sample size of our study inhibits our ability to define specific thresholds at which deer habitat selection and movement decisions change across these different environmental gradients (i.e. severity, land cover, and mountain lion encounter probability) or the specific strength of these responses. Despite this, we believe that our findings offer robust insights into the general relationship between deer behavior and these environmental variables within this specific ecosystem type that may guide more targeted studies in the future that are able to define these thresholds across different contexts.

During this study, we observed a preference for burned areas by black-tailed deer in the “First Spring” and “1 Year Post Fire” time periods, potentially highlighting some of the benefits of returning wildfire to fire adapted ecosystems. Whereas megafire is a more extreme example of fire disturbance, more moderate disturbances such as prescribed fire or managed wildfire are known to perform important ecological work in maintaining key ecosystem functioning for local communities [81] and generating improved habitat and resources for wildlife [82], without the more deleterious impacts created initially by megafire. These managed wildfire approaches also serve an important function in reducing the incidence of megafires by promoting landscape heterogeneity and reducing continuous fuel loads [83, 84]. Deer utilization of different habitat patches that have burned at different severities across different portions of the year also further supports the theorized importance of pyrodiversity in these landscapes as well [85]. Thus, utilizing fire management may simultaneously accomplish important wildlife conservation goals (habitat creation and maintenance) and wildfire management goals (megafire prevention) in similar fire-prone ecosystems.

We found evidence to suggest that deer are resilient to the impacts of megafire on a 1-year time scale, but more work is necessary to understand whether these initial responses translate into longer term resilience. The lagged effects of megafire may present novel challenges to species that have adapted to historical fire regimes by altering longer cycles in resource availability [86] as well as interspecies interactions [87]. For example, in oak woodland savannas where acorn masting is a primary food resource for many herbivorous species [88, 89], megafires that top-kill mature oak trees could dramatically alter the availability of these resources until oaks are able to regenerate and begin masting again. These indirect impacts could have powerful effects on future population dynamics through responses like individual fitness and reproduction across the previously burned landscape. Future work that examines how megafire influences the density and demographic trends of mule deer across longer time periods could help assess the resilience of the species across broader temporal scales and help define the potential consequences of megafire on longer-term interspecies interactions such as herbivory and predation.

## Conclusion

Black-tailed deer are known generally for their tendency towards non-plastic behaviors and high site fidelity to their home ranges and migration routes [29, 35]. Our study, however, revealed additional nuance in our understanding of deer behavior in response to sudden change. Despite having naturally high site fidelity in the region of our study, we found that this black-tailed deer population had a great deal of adaptive capacity to change their movement and behavior to respond to the impacts and eventual resources following megafire. Climate change and climatic disturbances (such as megafire) may have a more severe impact on species that are unable to adjust their behavior to accommodate sudden changes in their environments [90]. Resilience of dominant herbivores could help facilitate ecological resilience at broader trophic levels following disturbance. We can help facilitate and boost the natural resilience we observed of black-tailed deer and other ungulates through land and fire practices that promote the benefits of fire while simultaneously avoiding the immediate drawbacks of megafire. Identifying the mechanism by which these layers of resilience are produced would not be possible without uncovering the nuances of animal behavior that underly these observed responses.

### Electronic supplementary material

Below is the link to the electronic supplementary material.

Supplementary Material 1

## Data Availability

Deer GPS data and novel code generated and analyzed for this study are archived in the Dryad repository: Calhoun, Kendall et al. (2023), Movement behavior in a dominant ungulate underlies successful adjustment to a rapidly changing landscape following megafire, Dryad, Dataset, doi:10.6078/D12H83. Deer collar data are stored as a CSV spreadsheet of all GPS locations for each deer throughout the study period. We utilize novel code to perform the analyses of this project. Sentiel-2 satellite imagery (https://sentinel.esa.int/web/sentinel/missions/sentinel-2) was obtained and processed using Google Earth Engine (https://earthengine.google.com/) to create fire severity rasters. The Hopland Research and Extension Center Shapefile Boundary and water streams shapefile used to extract satellite imagery and calculate distance to water, respectively, was originally downloaded from Hopland REC (https://hrec.ucanr.edu).

## Abbreviations

HREC: Hopland Research and Extension Center
RSF: Resource Selection Function
HMM: Hidden Markov Movement Model
NDVI: Normalized Difference Vegetation Index
EVI: Enhanced Vegetation Index
