# Supplementary material for: Movement behavior in a dominant ungulate underlies successful adjustment to a rapidly changing landscape following megafire
Source: Mov Ecol. 2024 Jul 31;12:53. doi: 10.1186/s40462-024-00488-4 (PMC11293098; doi:10.1186/s40462-024-00488-4)
Supplement: Supplementary file 1 — Supplementary Material 1 [file 40462_2024_488_MOESM1_ESM.pdf]

Authors: Kendall L. Calhoun, Thomas Connor, Kaitlyn M. Gaynor, Amy Van Scoyoc, Alex McInturff, Samantha E.S. Kreling, Justin S. Brashares

Title: Movement behavior of a dominant ungulate underlies successful adjustment to a rapidly changing landscape following megafire

Journal Name: Movement Ecology

Additional File 1 - Additional Figures and Tables

Table S1 - Sample size and average home range size of deer (*Odocoileus hemionus columbianus*) collared at the Hopland Research and Extension Center, CA, USA before and after the Mendocino Complex Fire in 2018. The Mendocino Complex Fire burned on July 27th, 2018.

Time Period	***Dates***	***n (Number of Collared Deer)***	***Average Home Range Size (km^2)***	***Home Range Size SD***
Prespring	March 1, 2017 – May 1, 2017<br>March 1, 2018 – May 1, 2018	n = 5	0.24	±0.10
Prefire	May 25, 2018 – July 25, 2018	n = 7	0.18	±0.06
Recently Burned	August 1, 2018 – October 1, 2019	n = 9	0.40	±0.15
First Spring	March 1, 2018 – May 1, 2018	n = 11	0.49	±0.15
1 Year Post Fire	August 1, 2019 – October 1, 2019	n = 6	0.19	±0.07

Table S2 – Number of collected GPS points collected from each collared deer (*Odocoileus hemionus columbianus*) at the Hopland Research and Extension Center before and after the Mendocino Complex Fire in 2018. Percent (%) collected displays the percentage of GPS fixes recorded of the maximum possible (n = 1464).

Time Period	***Deer ID***	***# GPS Points***	***% Collected***
Prespring	A1	1390	94.95%
Prespring	B2	1432	97.81%
Prespring	C3 *(first deployment)*	1390	94.95%
Prespring	C3 *(second deployment)*	990	67.62%
Prespring	F5	574	39.21%
Prefire	D3	515	35.18%
Prefire	H2	535	36.54%
Prefire	I5	531	36.27%
Prefire	J1	517	35.31%
Prefire	J2	515	35.18%
Prefire	J3	517	35.31%
Perfire	Q5	533	36.41%
Recently Burned	D3	1456	99.45%
Recently Burned	H2	1459	99.66%
Recently Burned	I5	1442	98.50%
Recently Burned	J1	1458	99.59%
Recently Burned	J2	1464	100%
Recently Burned	J3	1463	99.93%
Recently Burned	K1	1454	99.32%
Recently Burned	K2	1445	98.70%

Recently Burned	Q5	1454	99.32%
First Spring	C1	1460	99.73%
First Spring	D3	1452	99.18%
First Spring	H2	1461	99.80%
First Spring	I5	1458	99.59%
First Spring	J1	1460	99.73%
First Spring	J2	1461	99.80%
First Spring	K1	1457	99.52%
First Spring	K2	1461	99.80%
First Spring	M5	1462	99.86%
First Spring	N1	1463	99.93%
First Spring	Q5	1459	99.66%
1 Year Post Fire	A1	1454	99.32%
1 Year Post Fire	C1	1243	84.90%
1 Year Post Fire	C3	1458	99.59%
1 Year Post Fire	N1	1271	86.82%
1 Year Post Fire	N2	1456	99.45%
1 Year Post Fire	N3	1450	99.04%

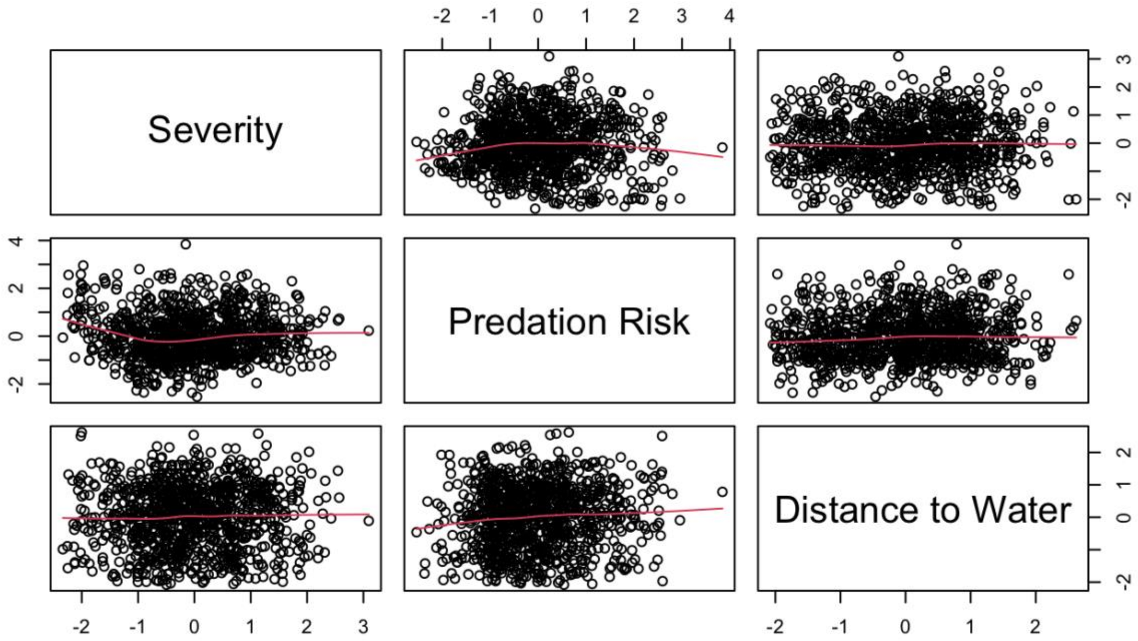

Figure S1 – Pairwise plots of continuous covariates extracted at the Hopland Research and Extension Center CA, USA used for deer resource selection function models and hidden Markov models. Plots were visually inspected to ensure there was no underlying covariance within models.

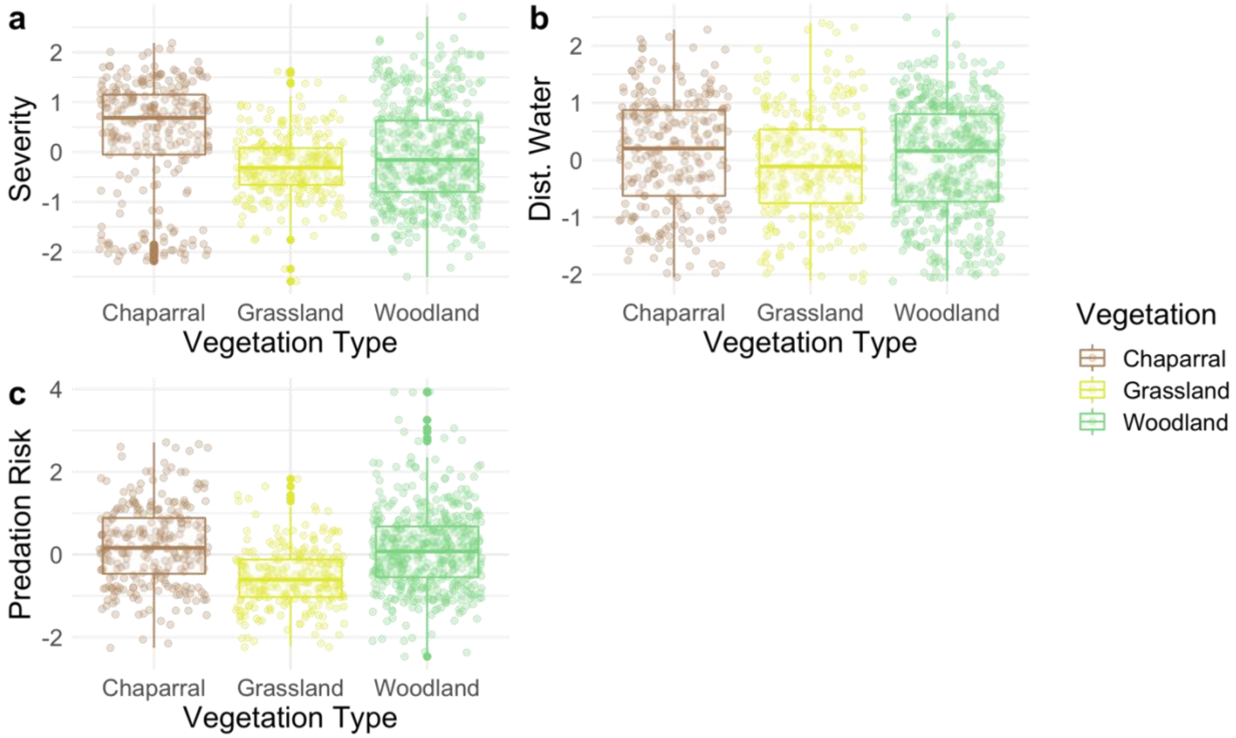

Figure S2 - Pairwise plots of continuous covariates extracted at the Hopland Research and Extension Center, CA, USA against categorical dominant vegetation types. These covariates were used for deer resource selection function models and hidden Markov models. Plots were visually inspected to ensure there was no underlying covariance within models.

Table S3 - Starting parameter value ranges for deer behavioral state estimation at the Hopland Research and Extension Center, CA, USA via the two-state hidden-Markov model (HMM). We estimated two behavioral states using the HMM: 1) Resting and 2) Traveling. We randomly selected values from within these ranges for each state in 25 model iterations. We compared the Maximum Likelihood across models to ensure they converged similarly and selected the starting parameters from the model that had the best fit in terms of maximum likelihood.

Parameter		***Resting***	***Traveling***
Step Length mean	min	0	400
	max	100	500
Step Length standard deviation	min	50	100
	max	75	200
Turning Angle mean		π	0
Turning Angle concentration	min	0.1	0.5
	max	0.5	3
Zero mass parameter		0.001040555	0.001040555

Table S4 - Welch's t-test results of home range size comparisons across different time periods before and after the 2018 Mendocino Complex Fire. The 2018 Mendocino Complex Fire burned through the Hopland Research Extension Center July 27, 2019. "Recently Burned" corresponds to deer home ranges estimated between August 1st, 2018 – October 1st, 2018. "First Spring" corresponds to home ranges estimated between March 1st, 2019 – May 1st, 2019. "1 Year Post Fire" corresponds to home ranges estimated between August 1st, 2019 – October 1st, 2019. "Prespring" corresponds to two combined springs seasons that occurred before the date of the fire: March 1st, 2017 – May 1st, 2017 and March 1st, 2018 – May 1st, 208. "Prefire" corresponds to deer home ranges estimated between May 25th, 2018 – July 25th, 2018. * denotes significant difference in home range estimates.

**Test**	**t**	**df**	**p-value**
**Recently Burned x First Spring**	-0.721	14.712	0.482
**Recently Burned x 1 Year Post Fire**	3.520	9.819	0.006*
**Recently Burned x Prespring**	2.656	9.481	0.025*
**Recently Burned x Prefire**	3.247	9.120	0.010*
**First Spring x 1 Year Post Fire**	5.976	13.945	0.001*
**First Spring x Prespring**	4.841	13.191	0.001*
**First Spring x Prefire**	5.746	12.765	0.001*
**1 Year Post Fire x Prespring**	- 2.008	8.998	0.076
**1 Year Post Fire x Prefire**	-0.833	9.8028	0.4247
**Prespring x Prefire**	1.396	8.787	0.197

Table S5 – Welch's t-test results of home range size comparisons using minimum number of GPS fixes (500 fixes per individual) for comparison. Five-hundred GPS fixes were randomly sampled from each deer within each time period to assess how robust our findings were to sample size (number of fixes). We find similar results in this rarefied example as our analysis using all the collected GPS-fixes. The 2018 Mendocino Complex Fire burned through the Hopland Research Extension Center July 27, 2019. "Recently Burned" corresponds to deer home ranges estimated between August 1st, 2018 – October 1st, 2018. "First Spring" corresponds to home ranges estimated between March 1st, 2019 – May 1st, 2019. "1 Year Post Fire" corresponds to home ranges estimated between August 1st, 2019 – October 1st, 2019. "Prespring" corresponds to two combined springs seasons that occurred before the date of the fire: March 1st, 2017 – May 1st, 2017 and March 1st, 2018 – May 1st, 208. "Prefire" corresponds to deer home ranges estimated between May 25th, 2018 – July 25th, 2018. * denotes significant difference in home range estimates.

Test	t	df	p-value
**Recently Burned x First Spring**	-0.639	13.261	0.534
**Recently Burned x 1 Year Post Fire**	3.198	9.689	0.010*
**Recently Burned x Prespring**	2.445	9.075	0.037*
**Recently Burned x Prefire**	3.101	8.799	0.013*
**First Spring x 1 Year Post Fire**	6.065	14.458	0.001*
**First Spring x Prespring**	5.055	13.108	0.001*
**First Spring x Prefire**	6.205	12.666	0.001*
**1 Year Post Fire x Prespring**	- 1.975	8.882	0.080
**1 Year Post Fire x Prefire**	-0.480	9.0206	0.643
**Prespring x Prefire**	1.866	8.789	0.096

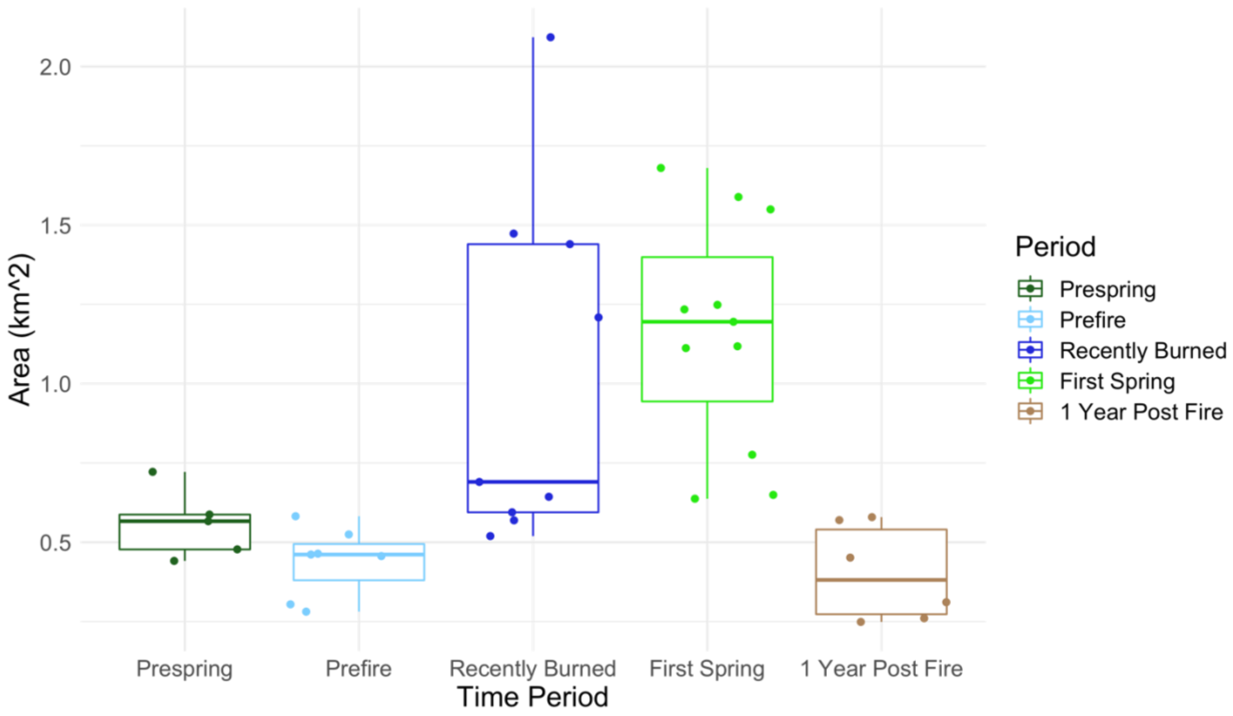

Figure S3 – Home range size of black-tailed deer (*O. hemionus columbianus*) across five time periods using minimum number of GPS fixes (n = 500) for comparison. Five-hundred GPS fixes were randomly sampled from each deer within each time period to assess how robust our findings were to sample size (number of fixes). We find similar trends in changes to homerange size in this rarefied example as our analysis using all the collected GPS-fixes. The Mendocino Complex Fire burned July 27th, 2018. These study periods include: 2017 Spring and 2018 Spring before the fire ("Prespring"), the summer season just before the fire burned ("Prefire"), directly following the fire ("Recently Burned"), the first spring following the fire ("First Spring"), and 1 full year post fire ("1 Year Post Fire") (*from left to right*).

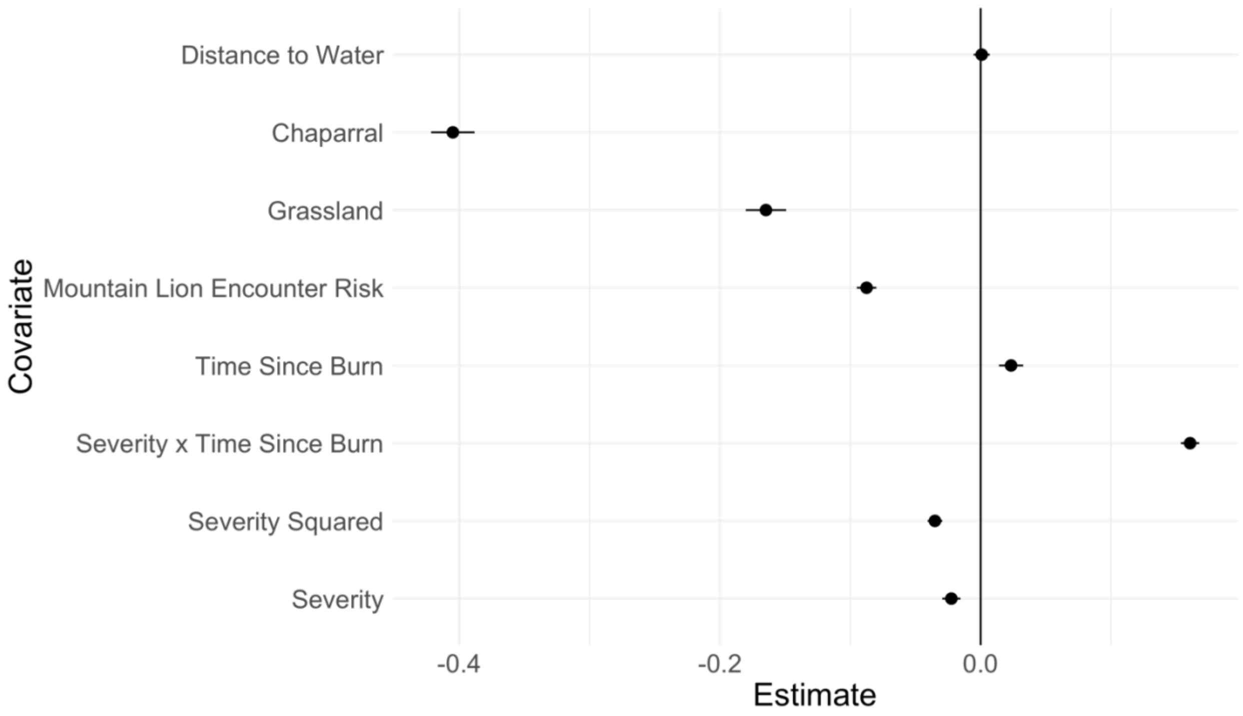

Figure S4 – Plotted beta coefficients of the Resource Selection Function model for black-tailed deer (*O. hemionus columbianus*) following the 2018 Mendocino Complex Fire at the Hopland Research and Extension Center, CA, USA. Associated standard error bars for each covariate are also plotted. All covariates to the left of the origin (0.0) are negatively associated with deer habitat selection all covariates to the right of the origin are associated with positive habitat selection. Covariates whose standard error ranges that do not overlap 0 were found to be statistically significant relationships.

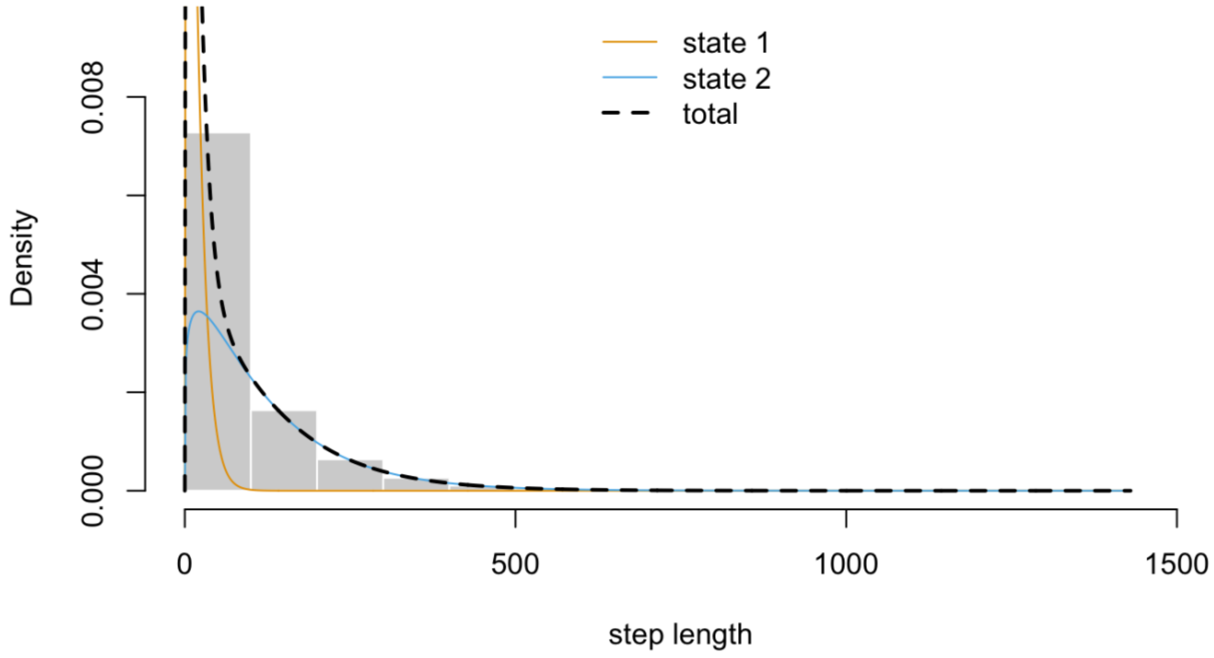

Figure S5 – Histogram of step lengths and density of each predicted state from the best fitting hidden Markov model for deer behavior at the Hopland Research and Extension Center, CA, USA. State 1 corresponds to “resting” and State 2 corresponds to “traveling”.

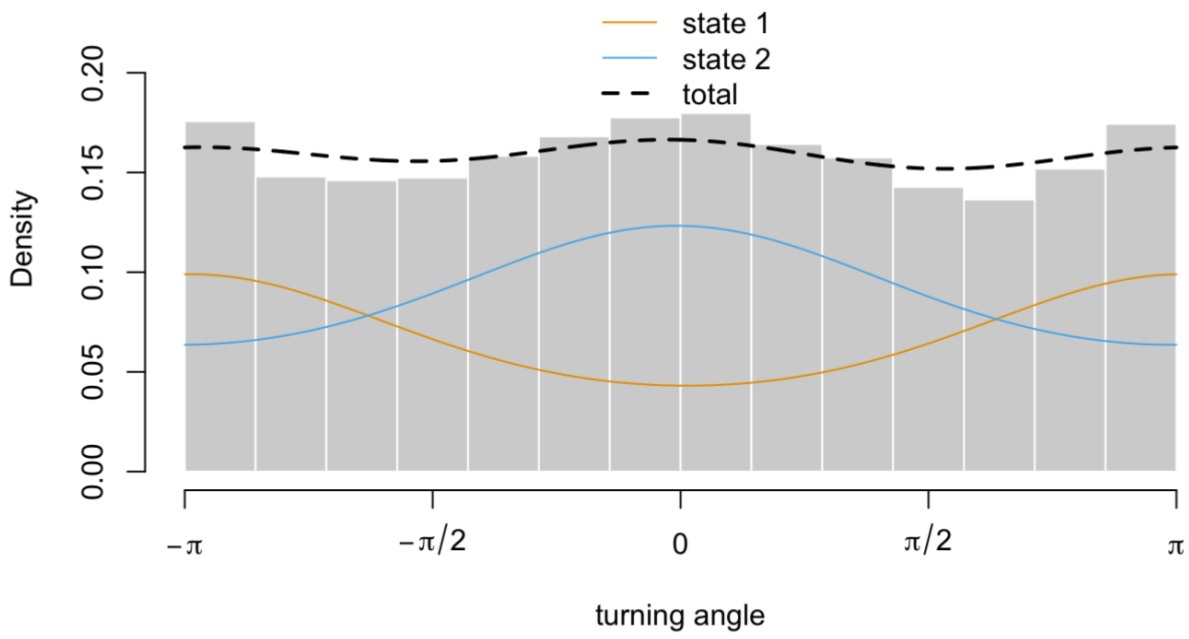

Figure S6 – Histogram of turning angles and density plots of each predicted state from the best fitting hidden Markov model for deer behavior at the Hopland Research and Extension Center, CA, USA. State 1 corresponds to “resting” and State 2 corresponds to “traveling”.

State	*Recently Burned*	*First Spring*	*1 Year Post Fire*
**1 (Resting)**	4558.304	6304.815	3767.693
**2 (Traveling)**	8553.696	9750.185	4562.307

Table S6 – Contingency table of deer behavioral states as estimated from the hidden Markov model for deer behavior at the Hopland Research and Extension Center. Behavioral states for each GPS-point were estimated using the "stationary" function of the "moveHMM" (v.1.8) package in R where State 1 = resting and State 2 = traveling. State probabilities for each GPS-point were then summed across within each Time Period (*Recently Burned, First Spring, or 1 Year Post Fire*).

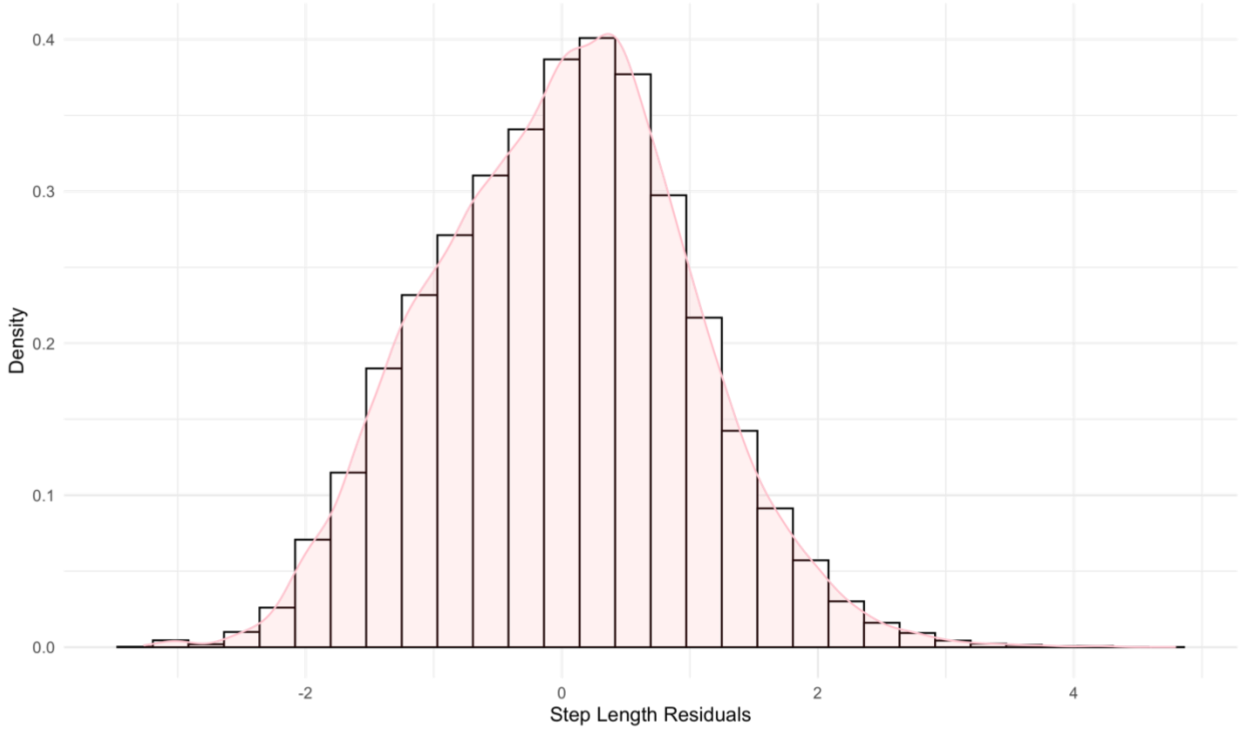

Figure S7 – Plotted histogram of step-length pseudo residuals from the fit hidden Markov movement model (HMM). The hidden Markov movement model was fit on deer GPS data following the 2018 Mendocino Complex Fire at the Hopland Research and Extension Center, CA, USA. We drew a random sample of pseudo-residuals from the fitted HMM to check goodness of fit of the model (n = 1000).

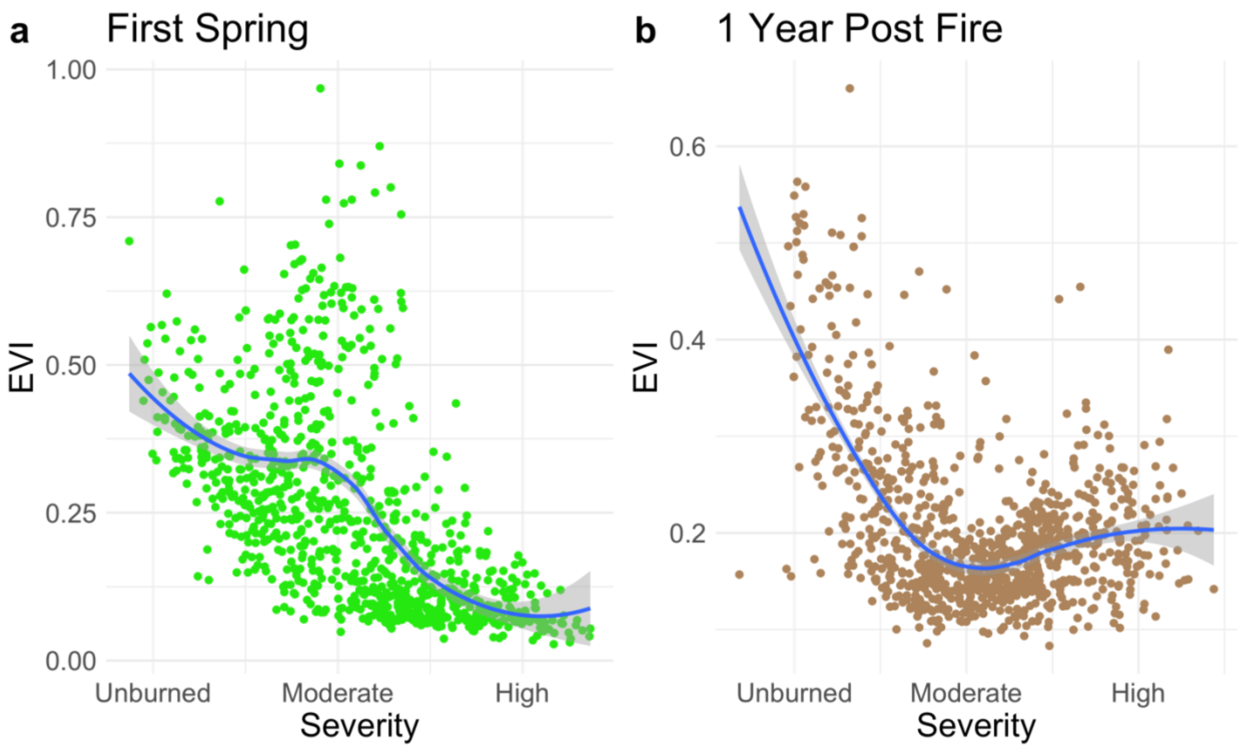

Figure S8 – Plotted relationship of forage quality, as represented by EVI (Enhanced Vegetation Index), and fire severity in the last two time periods of the study following the 2018 Mendocino Complex Fire at the Hopland Research and Extension Center, CA, USA. 1000 points were sampled across the study area and EVI and severity values were extracted at each point.
